# Tumor lysis syndrome, acute kidney injury and disease-free survival in critically ill patients requiring urgent chemotherapy

**DOI:** 10.1186/s13613-022-00990-1

**Published:** 2022-02-15

**Authors:** Moustafa Abdel-Nabey, Anis Chaba, Justine Serre, Etienne Lengliné, Elie Azoulay, Michael Darmon, Lara Zafrani

**Affiliations:** 1 Medical Intensive Care Unit, Saint-Louis University Hospital, Assistance Publique-Hôpitaux de Paris (AP-HP), 1 Avenue Claude Vellefaux, 75010 Paris, France; 2 University of Paris, Paris, France; 3 Hematology Department, Saint-Louis University Hospital, Assistance Publique-Hôpitaux de Paris (AP-HP), Paris, France; 4 INSERM UMR 976, University of Paris, Paris, France

**Keywords:** Tumor lysis syndrome, Acute kidney injury, Hematologic malignancy, Intensive care unit, Hemodialysis, Rasburicase

## Abstract

**Background:**

Tumor lysis syndrome (TLS) is a life-threatening complication during the treatment of malignant neoplasia. We sought to describe characteristics and predictors of acute kidney injury (AKI), remission and mortality in high-risk TLS patients. In this retrospective monocentric study, we included all patients with the diagnosis of biological and/or clinical TLS from 2012 to 2018. The primary outcome was the prevalence of AKI during the acute phase of TLS. Secondary outcomes were overall mortality and remission of the underlying malignancy at 1 year.

**Results:**

Among 153 patients with TLS, 123 (80.4%) patients experienced AKI and 83 (54.2%) required renal replacement therapy. mSOFA score (OR = 1.15, IC 95% [1.02–1.34]), age (OR = 1.05, IC 95% [1.02–1.08]) and male gender (OR = 6.79, IC 95% [2.59–19.44]) were associated with AKI. Rasburicase use (HR = 2.45, IC 95% [1.17–5.15]) was associated with remission of the underlying malignancy at 1 year. Parameters associated with mortality at 1 year were mechanical ventilation (HR = 1.96, IC 95% [1.02–3.78]), vasopressors (HR = 3.13, IC 95% [1.59–6.15]), age (HR = 1.02, IC 95% [1–1.03]), spontaneous TLS (HR = 1.65, IC 95% [1.01–2.69]) and delay of chemotherapy administration (HR = 1.01, IC 95% [1–1.03]).

**Conclusions:**

AKI is highly prevalent in TLS patients. Rasburicase is associated with better outcomes regarding remission of the underlying malignancy. As rasburicase may be an indirect marker of a high degree of tumor lysis and chemosensitivity, more studies are warranted to confirm the protective role of urate oxidase. Delaying chemotherapy may be deleterious in terms of long-term outcomes.

**Supplementary Information:**

The online version contains supplementary material available at 10.1186/s13613-022-00990-1.

## Background

Tumor lysis syndrome (TLS) is a life-threatening complication that may occur during the treatment of hematological malignancies or, less frequently, solid tumors. TLS occurs due to rapid destruction of tumor cells after cancer chemotherapy initiation, but also spontaneously in one-third of the cases [1, 2]. According to consensus definition, biological TLS is characterized by the rapid release of intracellular contents with subsequent 25% change or level above or below average, for any two or more serum values of uric acid, potassium, phosphate, and calcium within 3 days before or 7 days after the initiation of chemotherapy [3, 4].

In addition, high amounts of purine bases stemming from extracellular deoxyribonucleic acid (DNA) are converted into xanthine and then uric acid, which becomes highly concentrated in the blood compartment. In this context, acute kidney injury (AKI) negatively impacts complete remission and survival rates [5–7]. The primary reported mechanisms of TLS-induced AKI are crystal nephropathies due to the precipitation of uric acid, xanthine and/or calcium phosphate crystals in renal tubules [2]. Nevertheless, in this context, other mechanisms can lead to acute tubular necrosis such as the release of pro-inflammatory cytokines impacting renal microcirculation but also tubulointerstitial lesions via tumor infiltration of the renal parenchyma or ureteral compression by tumor mass [8, 9]. As defined by consensus definition, other clinical TLS manifestations are cardiac arrhythmia and seizures [3]. TLS sometimes mimics sepsis with systemic inflammatory response syndrome and multiple organ failures including acute respiratory failure and/or hemodynamic instability [10].

Early identification of high-risk TLS (HR-TLS) patients is of utmost importance to prevent the occurrence of clinical TLS [11]. Hydration with physiological serum and hypouricemic agents (allopurinol and rasburicase) are the cornerstone of TLS management. Rasburicase, a recombinant urate oxidase, metabolizes urate in allantoin, a compound ten times more soluble than uric acid [12]. Despite its large use, Darmon et al*.* [1] found an occurrence of TLS in two-thirds of HR-TLS patients with AKI in half of them. Moreover, data are scarce on the general management and outcome of TLS in adults, especially in the rasburicase era. Previous studies included only a small number of patients, and most of them excluded intensive care unit (ICU) patients. Here, we report a large retrospective cohort of ICU patients developing TLS. Our objectives were to report the prevalence of AKI in patients with TLS and to correlate acute-phase data with disease-free survival and mortality at 1 year.

## Methods

This is a retrospective single-center cohort study performed in a university hospital ICU. This study was approved by an Institutional Review Board (IRB) (“Comité d’Evaluation de l’Ethique des projets de Recherche Biomédicale Paris Nord”—IRB 00006477—of Paris 7 University). According to the French regulation, the need for informed consent was waived. Patients were informed that their data might be used for research purposes, and none refused. The study was conducted following the Declaration of Helsinki principles.

### Study population

We included consecutive patients admitted to the ICU of the Saint-Louis University hospital and having either biological and/or clinical TLS from January 2012 to July 2018. Patients were admitted to the ICU either from outside the hospital or from one of the 8 hematology wards in the hospital. The Saint-Louis University Hospital is a 700-bed public hospital with 483 beds for patients with hematologic malignancies and solid cancers. The ICU is a 12-bed medical unit that admits 950 patients per year, of whom about one-third have hematologic malignancies. Information on the organization of our ICU and criteria for ICU admission have been published elsewhere [13]. ICU admission policies remained unchanged throughout the study period. At our institution, senior hematologists and intensivists are available 24 h a day 7 days a week and work together to manage all high-risk hematology patients. Chemotherapy was prescribed by the hematologist in charge of the patient, according to the best standard of care [14, 15]. Due to the lack of data on the indication and timing of renal replacement therapy (RRT) initiation in this context, the choice was left to the clinician’s discretion in charge of the patient. Clinical, biological and imaging data were retrieved from medical records. Treatment and outcomes were also collected retrospectively. Assessment of complete remission was achieved according to recent criteria for each hematological malignancy [16].

### Definitions

Biological and clinical diagnosis was established according to consensus definition [3] (Additional file 1). The risk of TLS was assessed according to usual guidelines [4]. AKI was considered for an elevation of serum creatinine and/or a decreased urine output as defined by the KIDGO Clinical Practice Guidelines [17] within 7 days after the TLS. When available, the pre-admission serum creatinine available in a time period of a maximum of 1 year and a minimum of 7 days from the moment of hospital admission was considered as baseline. Baseline creatinine was only available in 86 (56%) patients. When baseline creatinine was unavailable (corresponding to 67 (44%) patients), we used the lowest creatinine measured during ICU hospitalization [18–21]. Estimated glomerular filtration rate (eGFR) was calculated by the CKD Epidemiology Collaboration equation. The clinical, laboratory and imaging data for each patient were reviewed by two nephrologists, who reached a consensus regarding AKI diagnosis.

As previously defined, the Sequential Organ Failure Assessment (SOFA) score was also recorded [22]. To avoid the potential burden of renal failure in the SOFA score, we used a modified SOFA (mSOFA) without renal criteria to assess the impact of organ failure on the occurrence of AKI.

The follow-up of patients with high-grade malignancies is performed every month and then every 3 months during the first year after induction chemotherapy. The status of remission, relapse, or refractory disease was defined by the hematologist in charge of the patient at 1 year.

### Outcomes

The primary outcome was the prevalence of AKI during the acute phase of TLS. Secondary outcomes were overall mortality and remission of the underlying malignancy at 1 year.

### Tumor lysis syndrome management

For the high-risk TLS or with biological TLS management, we followed the guidelines of Jones et al. [23]. As recommended, hydration status was assessed every day and hydration with 3 l/m^2^/24 h of saline serum was performed to maintain a urine output of 100 ml/m^2^/h. Balanced or isotonic solutions were used with no potassium addition to the hydration fluid. Urine output was measured every 3 h and a formal assessment of fluid balance was undertaken at least 6 hourly. We did not use diuretics in TLS patients unless there was pulmonary edema. There is a lack of studies concerning the indications and timing of RRT during TLS. RRT is started as recommended in untraceable fluid overload, hyperkalemia and rapidly increasing hyperphosphatemia. In our department, RRT is commonly started when phosphatemia is above 7.7 mg/dl or when phosphatemia increase is > 3 mg/dl every 6 h.

### Statistical analyses

Data are reported as absolute value with percentage for categorical variables or median with an interquartile interval for quantitative variables. A competing risk analysis was performed to assess the cumulative risk of remission and depict it. Concomitant competing risks considered were “discharged alive from the ICU” and “ICU mortality”. The time-dependent Cox model and Fine and Gray model were used to assess risk factors of remission at 1 year and one-year mortality. Logistic regression was used to assess risk factors of AKI after TLS.

Models were built using a conditional backward stepwise variable selection process based upon variable influence in univariate analysis. Critical entry and exit p values were 0.2 and 0.1, respectively. It was preplanned to force clinically relevant variables (TLS risk) into the final model if they were not previously selected. Correlation and interaction were carefully checked within final models as were checked assumption for log-linearity of continuous variables and proportional hazard assumptions for survival models. Data are given as odds ratios (OR, 95%CI), hazard ratios (HR; 95%CI), or sub-hazard ratios (sHR; 95%CI) according to the used model.

In a way to further explore the influence of rasburicase on mortality and remission, we performed a propensity score weighting analysis. Briefly, overlap weighting was performed. This strategy allows weighting patients from each treatment group with probability to be assigned to the other treatment group [24]. This allows assigning a higher weight to patients with intermediate-risk and lower weight to outliers in both treatment groups, the analysis emphasizing the proportion of the population where the most treatment equipoise exists in clinical practice [25]. This model has demonstrated high stability and the ability to obtain precise adjustment in various situations [26]. The propensity score was built using logistic regression according to variables associated with rasburicase and likely to have participated in to use of this treatment. Covariates included in the model were age, gender, underlying G6PD deficiency, underlying malignancy, HIV infection, severity, AKI and its severity and urates level at ICU admission. Quality of matching was assessed using propensity score distribution before and after weighting and variables distribution after weighting. The influence of rasburicase on mortality and remission was then assessed using Kaplan–Meier survival curve, cumulative incidence, and time-dependent Cox model.

To further assess the influence of outliers in our results, and as sensitivity analysis aiming to assess the robustness of our findings, we performed a complex bootstrap resampling [27]. Briefly, we used a bootstrapping technique, resampling the original set 1000 times with replacement then in each of the sets we assessed, for both mortality and the cumulative rate of remission, the unadjusted risk, the matched risk (propensity score matching like the one used in the first analysis with nearest neighbor technique) and last the weighted risk (overlap weighting like the previously described one).

Statistical significance was considered using two-sided tests with a critical alpha risk of 0.05.

Statistical analyses were performed using R version 3.6.2 (R Foundation for Statistical Computing), “survival”, “cmprisk”, “survey”, and “WeightIt” packages.

## Results

### Patients’ characteristics and outcome

**Fig. 1:**
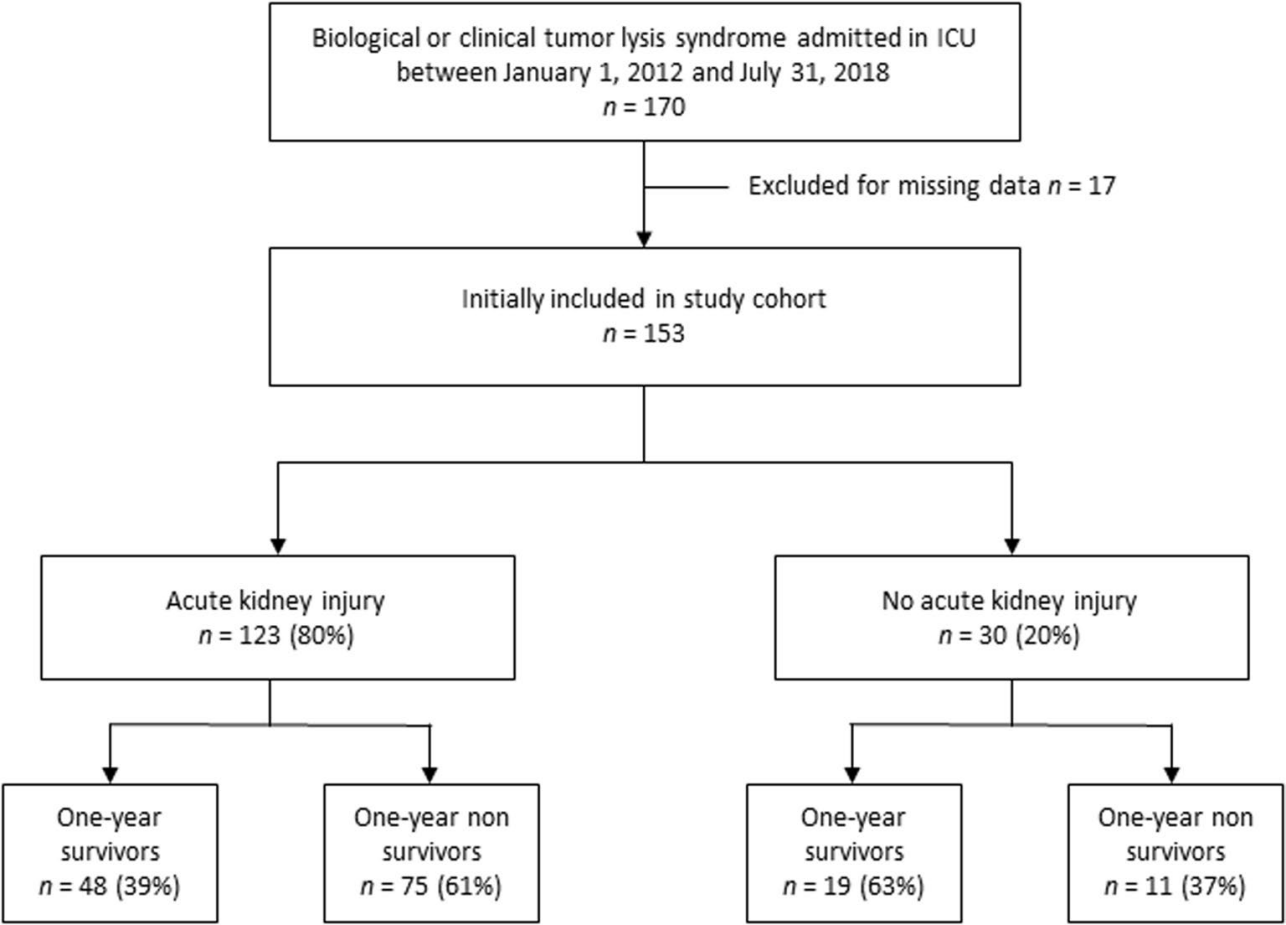
Study flowchart

Data of 170 patients were collected following TLS criteria. Seventeen patients were excluded because of missing data. The 153 remaining patients were included (Fig. 1). Baseline characteristics, clinical and laboratory data are shown in Table 1. The median age at diagnosis was 61 (46–69) years. Most of the patients had an underlying hematological malignancy. Among them, 52 (34%) patients had leukemia and 90 (59%) patients had non-Hodgkin lymphoma (NHL). One patient with TLS underwent cardiac arrhythmia and one had seizures. The overall mortality rate at 1 year was 56.2% (*n* = 86). By univariate analysis, non-survivors at 1 year presented more spontaneous TLS (65.1% versus 50.7%, *p* = 0.012), a higher SOFA score at admission (median = 7.5 versus 5, *p* < 0.001) and required more vasopressors (36% versus 1.5%, *p* < 0.001) and mechanical ventilation (33.7% versus 7.5%, *p* < 0.001) than survivors. There was no difference in terms of TLS risk and biological markers of TLS between survivors and non-survivors. Rasburicase has been administered in 60 (69.8%) of 1-year survivors versus 54 (80.6%) in non-survivors. Due to G6PD deficiency, 9 patients did not receive rasburicase. The first rasburicase dose was administered the day of ICU admission in 42 (36.8%) patients and 66 (57.9%) in the first 24 h of ICU admission. Only 6 patients received rasburicase before ICU admission. Clinical TLS was present in all patients on the day of admission. One administration was given in 114 (74.5%) patients at a dose of 7.5 mg, two administration in 46 (30.3%) and 3 in 11 (7.2%) patients. Allopurinol was used in 19 (12.5%) patients at intermediate risk of TLS (without hyperuricemia) and in high-risk patients with G6PD deficiency. None of the patients received febuxostat.

**Table 1:** Demographics details, clinical data, and laboratory data. Results are reported as n (%) or median (IQR)

	Death at 1 year		Total (*N* = 153)
	No (*N* = 67)	Yes (*N* = 86)	
Age (years)	60.00 (38.50–68.50)	63.00 (50.25–70.00)	61 (46–69)
Male	45 (67.2)	60 (69.8)	105 (69)
G6PD deficiency	7 (10.4)	2 (2.3)	9 (6)
Underlying malignancy			
Solid cancer	0 (0)	2 (2.3)	2 (1)
Leukemia	23 (34.3)	29 (33.7)	52 (34)
Non-Hodgkin lymphoma	38 (56.7)	52 (60.5)	90 (59)
Multiple myeloma	2 (3.0)	0 (0)	2 (1%)
Other	4 (6.0)	3 (3.5)	7 (5%)
Spontaneous TLS	34 (50.7)	56 (65.1)	90 (59)
Risk of TLS			
Low risk	9 (13.4)	15 (17.4)	24 (16)
Intermediate risk	5 (7.5)	4 (4.7)	9 (6)
High risk	53 (79.1)	67 (77.9)	120 (78)
Cardiovascular risk			
Chronic hypertension	19 (28.4)	27 (31.4)	46 (30.1)
Diabetes	7 (10.4)	12 (14.0)	19 (12.4)
Cardiovascular event	5 (7.5)	10 (11.6)	15 (9.8)
Chronic kidney disease	6 (9.2)	4 (4.7)	10 (6.6)
HIV positive status	7 (10.4)	19 (22.1)	26 (17)
SOFA score	5.00 (3.00–8.00)	7.50 (5.00–12.00)	6.5 (4–9)
Vasopressors	1 (1.5)	21 (36)	24 (16)
Mechanical ventilation	5 (7.5)	29 (33.7)	34 (22.2)
Rasburicase use	54 (80.6)	60 (69.8)	114 (75)
Laboratory data at admission			
Kalemia, mEq/L	4.60 (4.00–5.00)	4.30 (3.90–5.00)	4.3 (3.9–5.0)
Phosphatemia, mg/dL	4.59 (3.61–6.08)	4.19 (3.08–5.94)	4.45 (3.41–5.88)
Calcemia, mg/dL	8.94 (7.90–9.50)	8.50 (7.84–9.45)	8.80 (8–9.60)
Uricemia, mg/dL	8.96 (5.64–13.10)	7.49 (5.12–12.16)	8.46 (5.23–12.41)
LDH, UI/L	2700 (1544–5168)	2855 (1544–5263)	2819 (1533–5169)
Delay admission-chemotherapy (days)	1 (0–7)	4 (0.5–12)	2.5 (0–11)
ICU mortality	0 (0)	27 (31.4)	27 (18)
One-year remission	54 (80.6)	0 (0.0)	54 (35.3)

*G6PD* glucose 6 phosphate dehydrogenase, *TLS* tumor lysis syndrome, *HIV* human immunodeficiency virus, *LDH* lactate dehydrogenase, *ICU* intensive care unit

### Chemotherapy administration

The majority of patients (72%) received chemotherapy and/or corticoids during the week before ICU admission or ICU stay. The other patients received it during the month after the ICU stay. First chemotherapy was administered 4 days (0.5–12) after ICU admission in non-survivors, versus 1 day (0–7) in survivors (*p* = 0.05).

Ninety patients had non-Hodgkin lymphoma with 57 (63%) of them experiencing spontaneous TLS. The median (IQR) time of chemotherapy initiation was 5.5 days (1–9.5) in the spontaneous TLS group versus 14 days (1.0–20.0), (*p* = 0.092).

In the leukemia group (*n* = 52), 31 patients had spontaneous TLS. The combination of hydroxycarbamide with dexamethasone was the most used cytoreduction therapy (46.2% in spontaneous TLS group versus 36.8% in other patients). The median (IQR) time of chemotherapy initiation was 0 days (0.0–1.5) in the spontaneous TLS group versus 0.50 (0.0–3.75), *p* = 0.45, with day 0 representing the ICU admission.

### Characteristics and risk factors of AKI

One hundred and twenty-three patients (80.4%) experienced AKI during ICU stay including 71.5% (*n* = 88) of AKI stage III (Table 2). The pre-admission serum creatinine was available in 86 patients. At admission, the mean serum creatinine level was 1.4 mg/dL and urinary sediment was most often bland. Eighty-three (54.2%) patients required RRT, either intermittent hemodialysis (IHD) in 80.7% of the cases or continuous renal replacement therapy (CRRT) (Additional file 1: Table S2). The median peak of serum phosphate levels before RRT initiation was 7.13 mg/dl (5.98–8.84). The highest serum phosphate levels in AKI patients and increases of serum phosphate levels from phosphatemia at admission to the peak of phosphatemia are shown in Additional file 1: Table S3 and Additional file 1: Figure S1. There was no use of nephrotoxic chemotherapy except for 2 patients (1 patient received ifosfamide and 1 received methotrexate) (Additional file 1: Table S4). Other nephrotoxic drugs are detailed in Additional file 1: Table S5. In the survivor’s group, the serum creatinine level at 1 year was available for 61 patients with a median of 0.87 mg/L (IQR [0.62–1.03]).

**Table 2:** Demographics details, clinical data, and laboratory data according to the occurrence of acute kidney injury during ICU stay

	Acute kidney injury		*p*
	No (* N* = 30)	Yes (* N* = 123)	
Age (years)	48.50 (31.50–64.75)	63.00 (50.50–70.00)	0.004
Male	12 (40.0)	93 (75.6)	< 0.001
G6PD deficiency	1 (3.3)	8 (6.5)	0.819
Underlying malignancy			0.452
Solid cancer	0 (0)	2 (2.3)	
Leukemia	13 (43.3)	39 (31.7)	
Non-Hodgkin lymphoma	17 (56.7)	73 (59.3)	
Multiple myeloma	0 (0.0)	2 (1.6)	
Other	0 (0.0)	7 (5.7)	
Spontaneous TLS	12 (40.0)	78 (63.4)	0.033
Nephrotoxic use	6 (20.0)	48 (39.0)	0.082
Cardiovascular risk			
Chronic hypertension	5 (16.7)	41 (33.3)	0.118
Diabetes	2 (6.7)	17 (13.8)	0.449
Cardiovascular event	2 (6.7)	13 (10.6)	0.763
Chronic kidney disease	2 (6.7)	8 (6.6)	1.000
HIV positive status	1 (3.3)	25 (20.3)	0.051
mSOFA score	4.00 (2.00–4.75)	5.00 (2.00–7.00)	0.021
Vasopressors	1 (3.3)	23 (18.7)	0.073
Mechanical ventilation	2 (6.7)	32 (26.0)	0.041
Rasburicase use	24 (80.0)	90 (73.2)	0.592
Laboratory data at admission			
Kalemia, mEq/L	4.10 (3.70–4.70)	4.60 (4.00–5.00)	0.084
Phosphatemia, mg/dL	3.53 (2.82–4.37)	4.71 (3.49–6.18)	0.003
Calcemia, mg/dL	8.94 (8.17–9.48)	8.68 (7.80–9.44)	0.421
Uricemia, mg/dL	6.84 (5.38–9.32)	8.78 (5.17–13.10)	0.166
LDH, UI/L	2700 (2008–4630)	2855 (1466–5286)	0.925
ICU mortality	3 (10.0)	24 (19.5)	0.338
One-year mortality	11 (36.7)	75 (61.0)	0.028
One-year remission	16 (53.3)	38 (30.9)	0.036

Results are reported as *n* (%) or median (IQR)

*G6PD* glucose 6 phosphate dehydrogenase, *TLS* tumor lysis syndrome, *HIV* human immunodeficiency virus, *mSOFA* modified Sequential Organ Failure Assessment, *LDH* lactate dehydrogenase, *ICU* intensive care unit

By multivariate analysis, covariates associated with AKI were mSOFA score (OR = 1.15, IC 95% [1.02–1.34]), age (OR = 1.05, IC 95% [1.02–1.08]) and male gender (OR = 6.79, IC 95% [2.59–19.44]) (Table 3).

**Table 3:** Covariates associated with acute kidney injury by logistic regression

	Multivariate analysis		
	OR	95%CI	*p*
Age (per year)	1.05	(1.02–1.08)	< 0.001
Male gender	6.79	(2.59–19.44)	< 0.001
mSOFA (per point)	1.15	(1.02–1.34)	0.044
Uricemia at admission (per mg/dL)	1.03	(0.95–1.13)	0.42

*mSOFA* modified Sequential Organ Failure Assessment

### One-year outcome

**Fig. 2:**
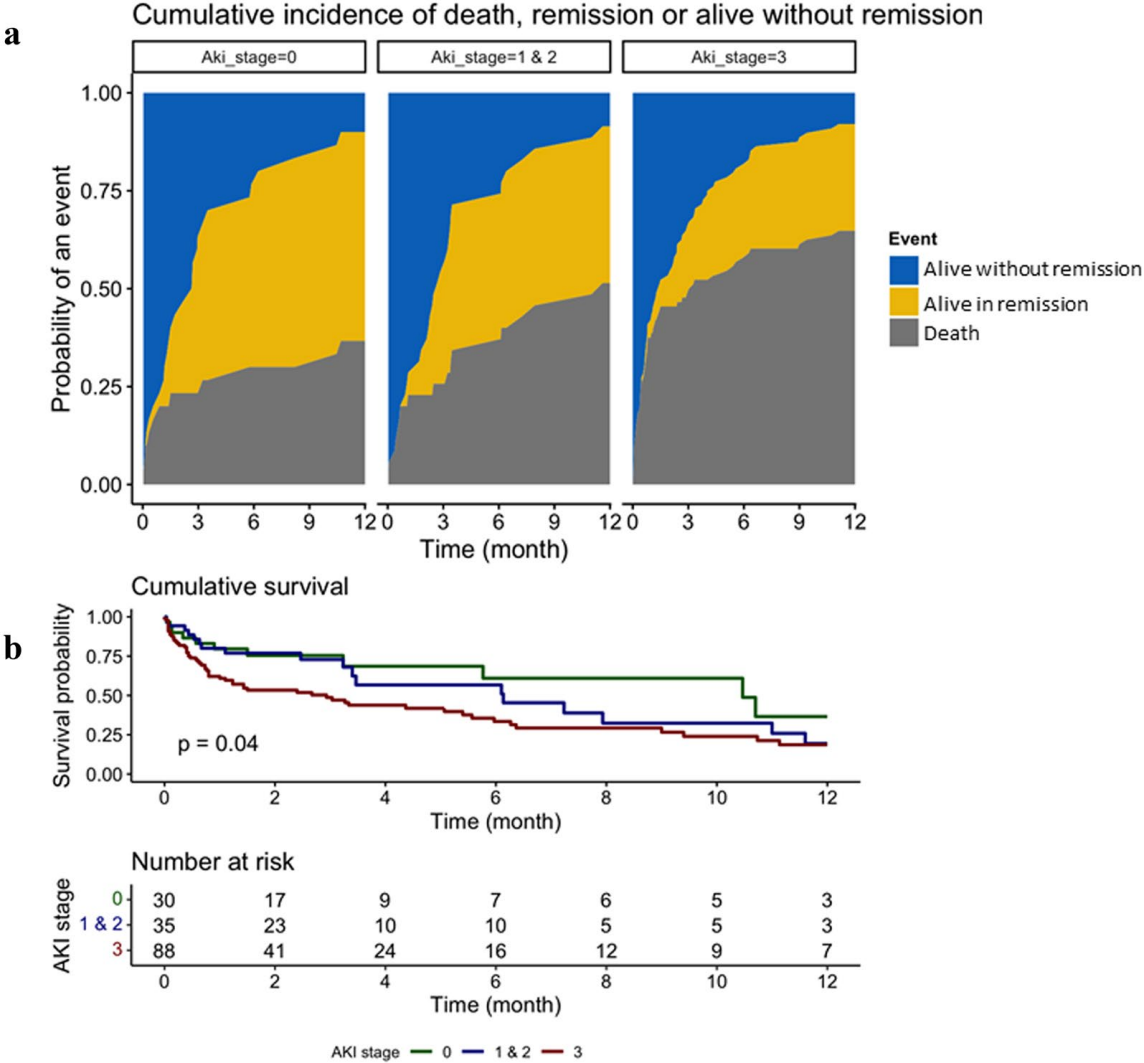
**a** Cumulative incidence of remission according to AKI and taking into account competing risks. **b** Kaplan–Meier survival estimates according to AKI stage

At 1 year, 54 patients (35.3%) were considered in remission of their hematological malignancy. By univariate analysis, AKI stage III was associated with mortality and less remission (Fig. 2). Finally, patients with AKI stage I–II seem to have a closer profile to patients without AKI in contrast to patients with AKI stage III (Fig. 2a and b).

By multivariate survival analysis, rasburicase use (HR = 2.45, IC 95% [1.17–5.15]) was associated with remission of the underlying malignancy at 1 year, independently of the initial risk of TLS. Vasopressors were associated with less remission at 1 year (HR = 0.12, IC 95% [0.01–0.98]) (Table 4).

**Table 4:** Covariates associated with remission at one year by Fine and Gray

	Multivariate analysis		
	sHR	95%CI	*p*
Mechanical ventilation	0.61	(0.22–1.72)	0.35
Vasopressors	0.12	(0.01–0.98)	0.04
Acute kidney injury	0.69	(0.37–1.26)	0.23
High risk of TLS	1.57	(0.78–3.16)	0.20
Rasburicase use	2.45	(1.17–5.15)	0.02

*TLS* tumor lysis syndrome

Parameters associated with mortality at 1 year were mechanical ventilation (HR = 1.96, IC 95% [1.02–3.78]), vasopressors (HR = 3.13, IC 95% [1.59–6.15]), age (HR = 1.02, IC 95% [1–1.03]), spontaneous TLS (HR = 1.65, IC 95% [1.01–2.69]) and delay of chemotherapy administration after ICU admission (HR = 1.01, IC 95% [1–1.03]) (Table 5).

**Table 5:** Covariates associated with death at one year by Cox survival analysis

	Multivariate analysis		
	HR	95%CI	*p*
Mechanical ventilation	1.96	(1.02–3.78)	0.04
Vasopressors	3.13	(1.59–6.15)	0.001
Acute kidney injury	1.09	(0.55–2.18)	0.80
Spontaneous TLS	1.65	(1.01–2.69)	0.04
Age (per year)	1.02	(1–1.03)	0.03
Delay admission-chemotherapy (per day)	1.01	(1–1.03)	0.04
Rasburicase use	1.08	(0.65–1.79)	0.76

*TLS* tumor lysis syndrome

AKI was not associated by multivariate analysis with remission or mortality

**Fig. 3:**
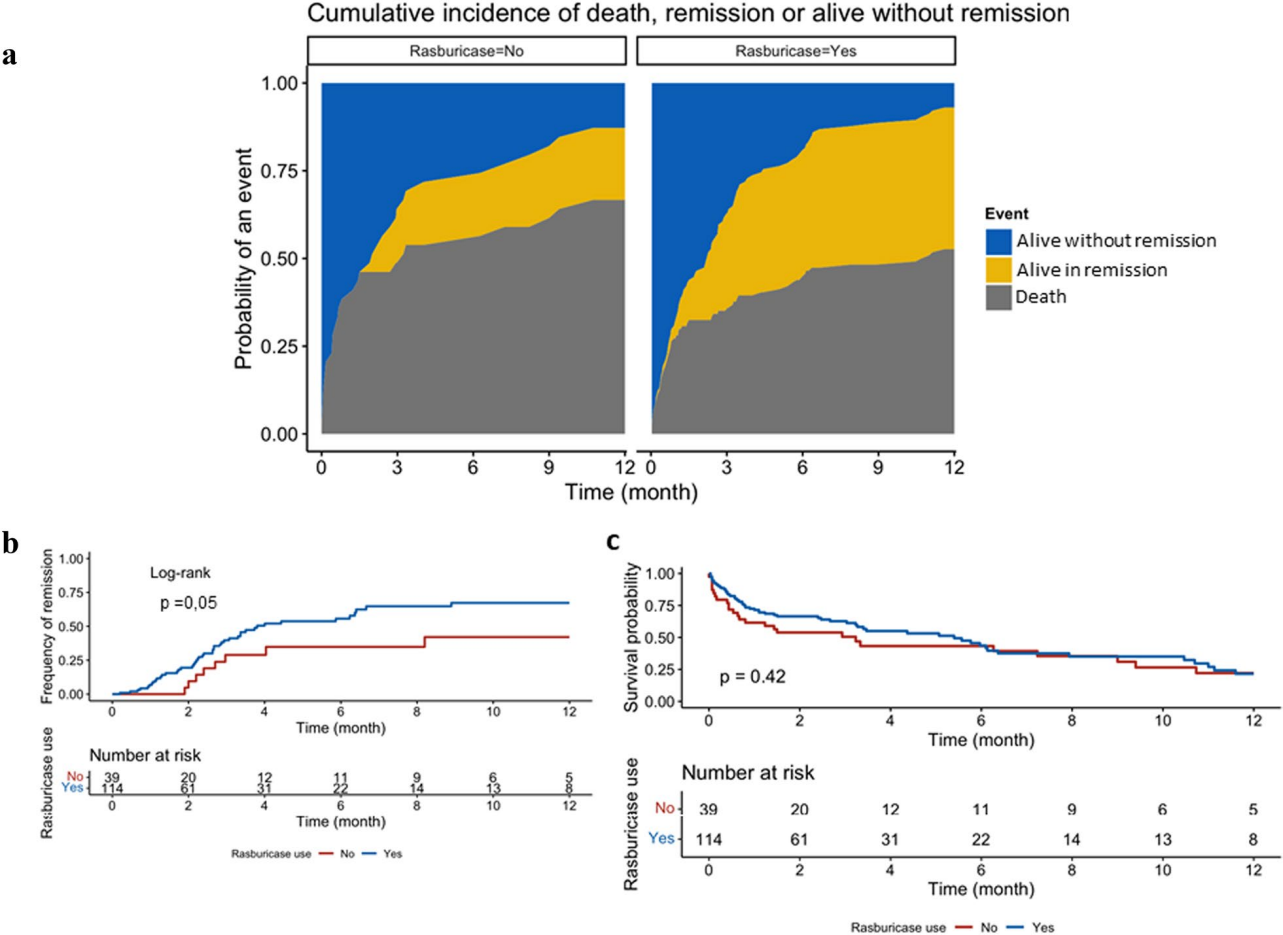
Outcome according to use of rasburicase before adjustment. **a** Cumulative incidence of remission according to rasburicase use and taking into account competing risks. **b** Cumulative incidence of remission according to rasburicase use. **c** Kaplan–Meier survival estimates according to rasburicase use

**Fig. 4:**
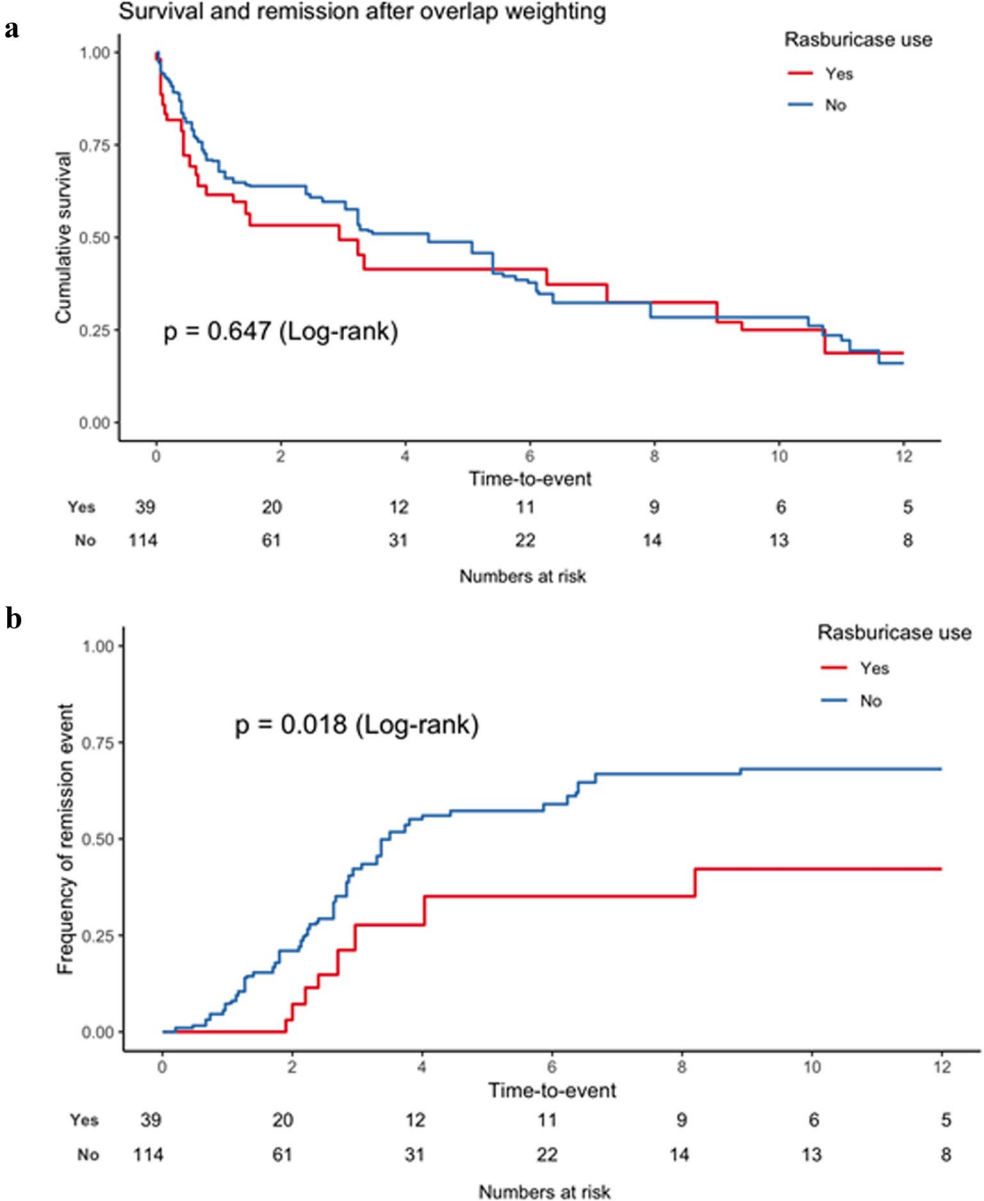
Outcome according to use of rasburicase after propensity score weighting. **a** Kaplan–Meier survival estimates according to rasburicase use. **b** Cumulative incidence of remission according to rasburicase use

To confirm the association between rasburicase use and remission, we performed a propensity score weighting. After weighting, both groups and propensity score were adequately balanced (Additional file 1: Figures S2 and S3). Rasburicase was associated with increased remission rate (Figs. 3a, b and 4b) but not with 1 year mortality (Figs. 3c and 4a).

Among the 67 survivors, renal outcomes were available for 49 patients. Median (IQR) GFR was 85 ml/min/1.73 m^2^ (80–113) in the non-AKI group and 78 ml/min/1.73 m^2^ (57.25–104.25) in the AKI group, *p* = 0.26. In the survivor group, among those who had AKI, 9 (26.5%) patients had a GFR below 60 ml/min/1.73 m^2^ versus 1 (6.7%), *p* = 0.23 (Additional file 1: Figure S4).

### Robustness of findings

Results from bootstrapping analysis were in line with the main results suggesting our findings may not be ascribable to outliers (Additional file 1: Figure S5).

## Discussion

To the best of our knowledge, this is the largest study describing general management and outcomes of high-risk TLS patients. These patients are more often excluded from trials or are included in studies classically focused on preventing AKI [28–30].

As previously shown by Darmon et al*.* [5], we found that AKI stage III was associated with poor outcomes (less remission of the underlying malignancy and survival) in univariate analysis. AKI requiring RRT may lead to sub-optimal chemotherapy administration and decrease the chances of optimally effective chemotherapy, even when the complete regimen is given. One possible explanation may pertain to the lack of knowledge on drug pharmacokinetics in renal dysfunction patients or under RRT. However, AKI was not associated with less remission or mortality by multivariate analysis.

In our cohort, AKI was highly prevalent at admission, occurring in 80% of the patients. These results may be explained by the majority of patients at high risk of TLS in our cohort (77%) in a selected ICU cohort. However, in our hospital, hematologists and intensivists work together to manage patients at high risk of TLS with an early policy of admission in ICU before the occurrence of organ failures.

One-third of the patients were considered in remission at 1 year. Rasburicase use and high risk of TLS were associated with higher remission rates. By performing three different analyses (propensity score weighting using overlap weights, propensity-matched analysis, and sensitivity analysis), we did not find any confounding factors in our data that could explain these results. The association of rasburicase with remission of the underlying malignancy may be explained by a protective effect of rasburicase on renal function, allowing optimal oncological treatment administration. Indeed, rasburicase prevents uric acid nephropathy and may improve renal outcomes. [31]. However, if rasburicase has been shown to be efficient in rapidly decreasing uricemia, its benefit in terms of renal outcomes and mortality are unclear [32]. Although rasburicase was a protective factor for remission independently of the initial risk of TLS at ICU admission in the multivariate analysis, rasburicase use may rather be the marker of a high degree of tumor lysis and chemosensitivity that could explain a better rate of remission of the underlying malignancy. In our cohort, we did not found that rasburicase administration was predictive of AKI occurrence. Since rasburicase is commonly used in TLS patients, the question of calcium phosphate crystals that may precipitate into the tubules is raised. However, the evidence for calcium phosphate crystals during TLS relies on scarce data from relatively old case reports [33, 34] with no available data estimating the prevalence of calcium phosphate crystals in urine patients, which led us to question the role of additional crystal-independent mechanisms in the pathophysiology of TLS-induced AKI. Nevertheless, uric acid has shown potential deleterious renal effects beyond crystal-induced mechanisms by inducing renal vasoconstriction and increasing renal oxidation and inflammation [35, 36]. According to our results, Pui et al. documented decreases in creatinine levels among 131 patients at high risk for TLS and were treated with rasburicase [37]. Canet et al*.* showed that the plasma uric acid decrease after rasburicase was significantly larger in patients who did not develop AKI than in those who did**.** Accordingly, we did not find any benefit of rasburicase in terms of mortality in our study. Hydration remains the cornerstone of TLS prophylaxis and treatment in these patients. Maintaining a high urine output with hydration and careful monitoring of fluid balance is crucial to prevent uric acid crystallization [4, 38, 39].

Other factors, such as spontaneous TLS, mechanical ventilation and vasopressors were associated with mortality. Spontaneous TLS before the administration of chemotherapy is usually a marker of high proliferative and extensive malignancies with a large tumor burden, explaining the higher mortality in these patients. Interestingly, delayed chemotherapy after ICU admission was also associated with increased mortality, independently of organ failures. This suggests that prompt chemotherapy should always be considered by intensivists and hematologists when managing these patients with high tumor burden.

This study also has several limitations. First, this is a retrospective study with inherently associated bias, such as unknown confounding factors that may have been overlooked in the data collection. Second, this study was conducted in a single-center, raising concern about the generalizability of our results. However, the large number of hematological patients managed in the different departments of our institution and the standardized policy of early ICU admission suggests that our results may apply to other settings. Third, due to the lack of histological data in these patients with a high risk of bleeding, the mechanisms of AKI are uncertain and may be multifactorial in the ICU setting and not directly the consequence of TLS. Many causes of AKI may indeed coexist, including hemodynamic instability, nephrotoxic use, mechanical ventilation, specific infiltration of the kidney and TLS per se. Moreover, fluid overload and venous congestion are essential factors that may participate to AKI. Finally, we could not distinguish among patients with AKI stage III those who had been dialyzed because of TLS but who would not have required RRT in other settings and precise data on phosphatemia kinetics before RRT initiation were unavailable. Indeed, in the absence of life-threatening metabolic disturbances, the optimal timing for initiating RRT in TLS-induced AKI is unknown. There are no guidelines concerning the threshold that should be used to start RRT. To this day, randomized control studies are lacking to prove the benefit of early RRT in this population.

## Conclusions

In high-risk patients, AKI is prevalent during TLS. Rasburicase and high risk of TLS were found to be associated with higher remission rates of the underlying malignancies. As rasburicase may be an indirect marker of a high degree of tumor lysis and chemosensitivity, more studies are warranted to confirm the protective role of urate oxidase. Delaying chemotherapy may be deleterious in terms of long-term outcomes.

## Supplementary Information

**Additional file 1.** Additional figures and tables.

## Data Availability

The datasets used and/or analyzed during the current study are available from the corresponding author on reasonable request.

## Abbreviations

AKI: Acute kidney injury
CRRT: Continuous renal replacement therapy
DNA: Deoxyribonucleic acid
eGFR: Estimated glomerular filtration rate
HR-TLS: High-risk TLS
ICU: Intensive care unit (ICU)
IRB: Institutional Review Board
mSOFA: Modified SOFA
NHL: Non-Hodgkin lymphoma
OR: Odds ratios
RRT: Renal replacement therapy
sHR: Sub-hazard ratios
SOFA: Sequential Organ Failure Assessment
TLS: Tumor lysis syndrome
