# Supplementary material for: Tumor lysis syndrome, acute kidney injury and disease-free survival in critically ill patients requiring urgent chemotherapy
Source: Ann Intensive Care. 2022 Feb 15;12:15. doi: 10.1186/s13613-022-00990-1 (PMC8847484; doi:10.1186/s13613-022-00990-1)

Additional data

Number of Table: 5

Number of Figures: 5

Table S1. Cairo–Bishop deﬁnition of laboratory and clinical tumor lysis syndrome in adult

Table S2. Renal findings

Table S3. Maximum serum phosphate levels

Table S4. Chemotherapy regimen

Table S5: Nephrotoxic drugs

Figure S1. Peak of phosphatemia in patients with or without AKI

Figure S2. Distribution balance of propensity score

Figure S3. Proportional balance of propensity score and Covariate balance

Figure S4. Evolution of Glomerular Filtration Rate over time

Figure S5. Bootstrapping analysis of the influence of the influence of rasburicase on mortality and remission

| Table S1. Cairo–Bishop deﬁnition of laboratory and clinical tumor lysis syndrome in adult | |
| --- | --- |
| Laboratory TLS  At least two if the following | |
| Uric acid | ≥ 8 mg/dL or 25% increase from baseline |
| Potassium | ≥ 6.0 mEq/L or 25% increase from baseline |
| Phosphorous | ≥ 4.5 mg/dL or 25% increase from baseline |
| Calcium | < 7 mg/dL or 25% decreases from baseline |
| Clinical TLS  Laboratory TLS as defined above plus at least one of the following | |
| Serum creatinine* ≥ 1.5 Upper limit of normal | |
| Cardiac arrythmia/sudden death* | |
| Seizure* | |

TLS : Tumor lysis syndrome

*Not directly or probably attribuTable Sto a therapeutic agent.

Table S2. Renal findings

| Variable | Overall (n=153) |
| --- | --- |
| Clinical |  |
| Kidney infiltration by malignant cells, n (%) | 18 (12) |
| Kidney size, cm, median (IQR) | 12.5 (10 - 14) |
| Urine output during the first 24h, median (IQR)  AKI stage (KDIGO), n (%) | 400 (0 - 500)  123 (80) |
| 0 | 30 (20) |
| 1 | 24 (16) |
| 2 | 11 (7) |
| 3 | 88 (58) |
| Renal replacement therapy, n (%) | 83 (54) |
| CRRT | 5 (6) |
| CRRT and IHD | 11 (13) |
| IHD  Hydration, L, median (IQR) | 67 (81)  3 (2-3) |
| Laboratory data, median (Interquartile range) |  |
| Baseline serum creatinine, mg/dL | 0.91 (0.74 – 1.05) |
| Baseline eGFR, ml/min/1.73m^2^ | 85 (71 - 99) |
| Serum creatinine at admission, mg/dL | 1.40 (0.96 – 2.23) |
| Serum urea at admission, mg/dL | 34.16 (22.4 – 50.4) |
| Proteinuria at admission, g/24h | 0.5 (0.2 - 1) |

AKI : Acute kidney injury ; CRRT: continuous renal replacement therapy; IHD: Intermittent hemodialysis

Table S3. Maximum serum phosphate levels

|  | Acute kidney injury | |  |
| --- | --- | --- | --- |
| Variable | No (N= 30) | Yes (N= 123) | pvalue |
| Phosphatemia peak pre RRTmedian (mg/dl) (IQR) |  | 7.13 (5.98 - 8.84) | NA |
| Phosphatemia peak, median (mg/dl) (IQR) | 4.96 (4.50 - 6.42) | 6.62 (5.60 - 8.26) | 0.002 |
| Admission phosphatemia/ peak phosphatemia ratio, median (IQR) | 1.29 (1.17 - 1.72) | 1.34 (1.08 - 1.76) | 0.953 |

Table S4. Chemotherapy regimen

| Chemotherapy | n(%) |
| --- | --- |
| **Leukemia,** | 52 (34) |
| ATRA/Antracycline/Cytarabine | 2 ( 4.4) |
| Clofarabine-Aracytine | 1 ( 2.2) |
| Cytarabine | 1 ( 2.2) |
| Cytarabine/Antracycline | 14 (31.1) |
| Cytarabine/Vindesine | 1 ( 2.2) |
| Endoxan/Doxorubicine/Daratumumab | 1 ( 2.2) |
| Endoxan/Nelarabine | 1 ( 2.2) |
| Etoposide/Aracytine | 1 ( 2.2) |
| GRALL | 3 ( 6.7) |
| Hydroxycarbamide | 19 (42.2) |
| VANDA | 1 ( 2.2) |
| **Non Hodgkin Lymphoma** | 90 (59) |
| CHOEP | 1 ( 1.2) |
| CHVP16 | 2 ( 2.5) |
| COP | 12 (15.0) |
| Cytarabine | 1 ( 1.2) |
| DHAX | 1 ( 1.2) |
| Endoxan | 9 (11.2) |
| Endoxan/Cytarabine | 1 ( 1.2) |
| Endoxan/Vincristine | 1 ( 1.2) |
| EPOCH | 1 ( 1.2) |
| Etoposide/Cytarabine | 1 ( 1.2) |
| Etoposide/Endoxan | 4 ( 5.0) |
| Etoposide/Endoxan/Doxorubicine | 3 ( 3.8) |
| ACVBP | 1 ( 1.2) |
| Ibrutinib | 1 ( 1.2) |
| R-ACVBP | 8 (10.0) |
| R-CHOP | 10 (12.5) |
| R-COP | 3 ( 3.8) |
| R-COPADEM | 2 ( 2.5) |
| R-DHAX | 5 ( 6.2) |
| R-EPOCH | 3 ( 3.8) |
| R-miniCHOP | 5 ( 6.2) |
| R-Mitoxantrine/Ifosfamide/VP16) | 1 ( 1.2) |
| R | 1 ( 1.2) |
| R-Chlorambucil | 1 ( 1.2) |
| R-Oxaliplatine/Cytarabine | 1 ( 1.2) |
| **Myeloma** | 2 (1) |
| R-Velcade/dexamethasone  **Solid cancer** | 1 ( 50)  2 (1) |
| Paclitaxel | 1 (50) |
| **Others** | 7 (4) |
| Fludarabine-Melphalan | 1 (16.7) |
| R-Bendamustine | 2 (33.3) |
| Venetoclax | 1 (16.7) |
| ***Abbreviations :***  *R : Rituximab*  *GRALL : vincristin-daunorubicin-prednisone-cyclophosphamide-L-asparaginase*  *ACVBP : doxorubicin-cyclophosphamide-vindesine-bleomycin-prednisone*  *CHOP : cyclophosphamide-adriamycine-vincristin-prednisone*  *CHVP16 : cyclophosphamide-adriamycine-etoposide*  *COPADEM :, cyclophosphamide- vincristine-doxorubicine, prednisone- méthotrexate*  *EPOCH : etoposide-erednisone-vincristine-cyclophosphamide- doxorubicin*  *DHAX : dexamethasone-cytarabine- oxaliplatin*  *VANDA : dexamethasone – cytarabine-mitoxantrone- etoposide L-Asparaginase* |  |

Table S5 : Nephrotoxic drugs

|  | No acute kidney injury  (n = 30) | Acute kidney injury (n = 123) |
| --- | --- | --- |
| Overall nephrotoxic product, n (%) | 6 (20.0) | 48 (39.0) |
| IV ICP n (%) | 4 (13.3) | 33 (26.8) |
| Vancomycin, n (%) | 1 (3.3) | 9 (7.3) |
| NSAID, n (%) | 1 (3.3) | 5 (4.1) |
| Aminoglycoside, n (%) | 1 (3.3) | 15 (12.2) |
| Nephroxic products combination, n (%) |  |  |
| NSAID alone | 1 (3.3) | 2 (1.6) |
| NSAID/IV ICP | 0 (0.0) | 1 (0.8) |
| NSAID/IV ICP/Aminoglycoside | 0 (0.0) | 2 (1.6) |
| Aminoglycoside alone | 0 (0.0) | 6 (4.9) |
| Aminoglycoside/Vancomycin | 0 (0.0) | 4 (3.3) |
| IV ICP alone | 3 (10.0) | 26 (21.1) |
| IV ICP/Aminoglycoside | 1 (3.3) | 2 (1.6) |
| IV ICP/Vancomycin | 0 (0.0) | 1 (0.8) |
| IV ICP/Vancomycin/Aminoglycoside | 0 (0.0) | 1 (0.8) |
| Vancomycin alone | 1 (3.3) | 3 (2.4) |

IV ICP: Intravenous iodinated contrast product; NSAID: Nonsteroidal anti-inflammatory drug

**Figure S1**.**Peak of phosphatemia in patients with or without AKI**
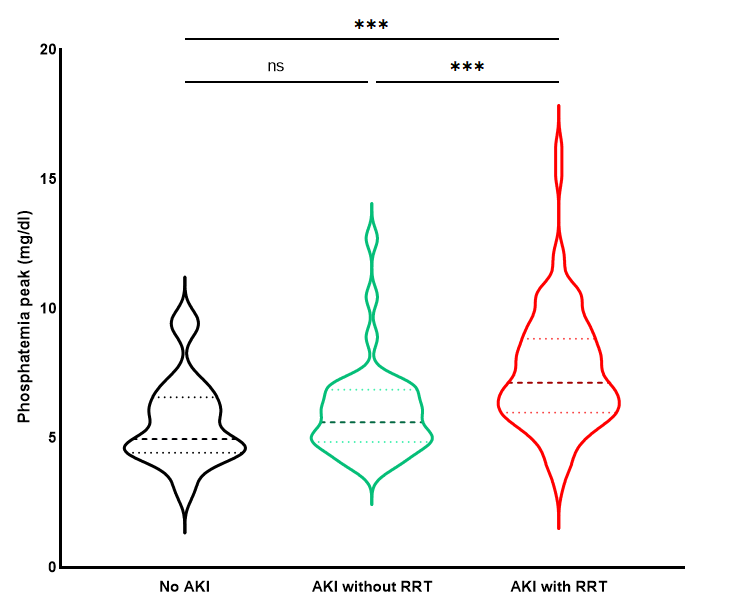


Figure S2. Distribution balance of propensity score


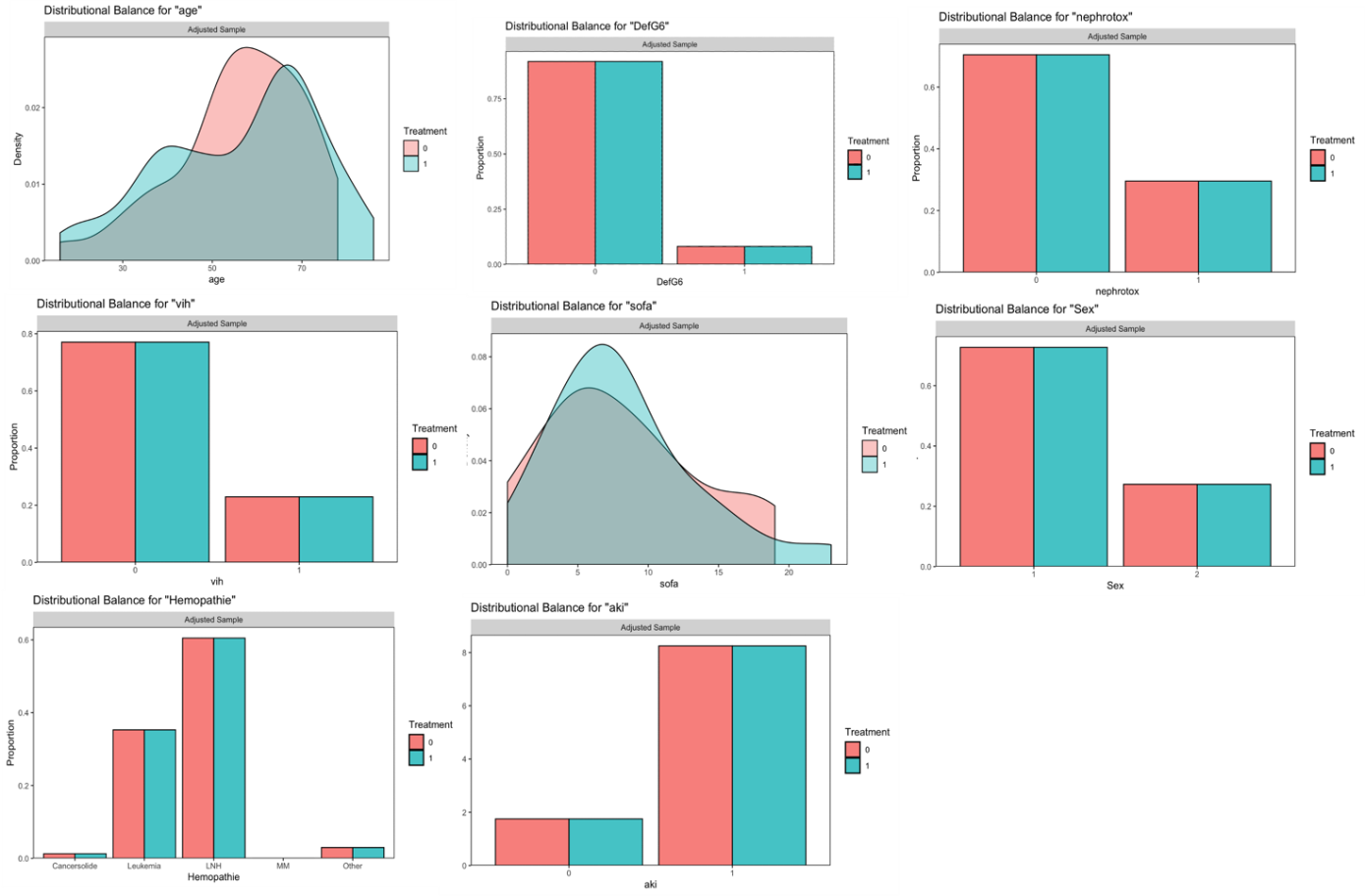


Figure S3. Proportional balance of propensity score and Covariate balance
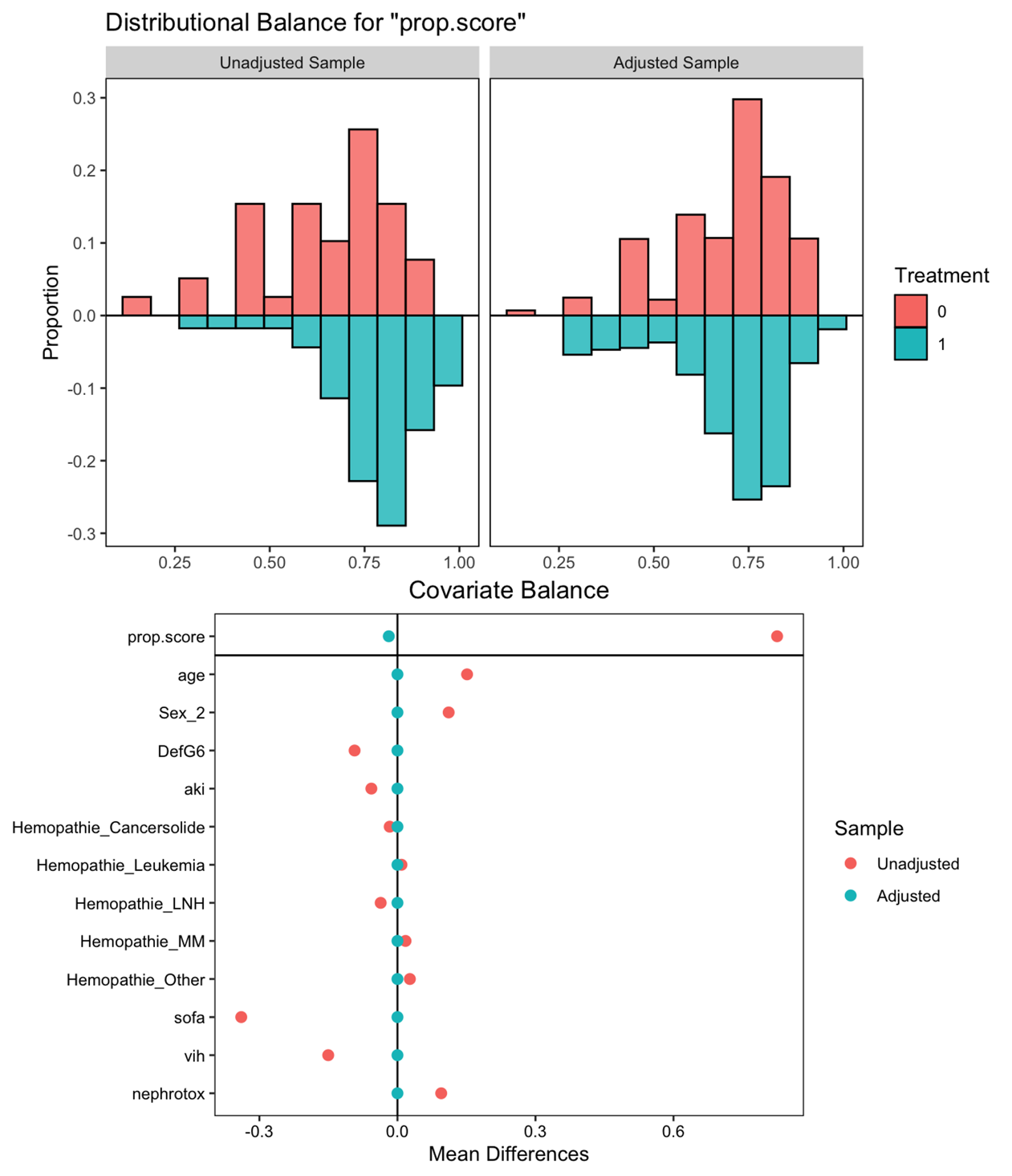


**Figure S4. Evolution of glomerular filtration rate over time**
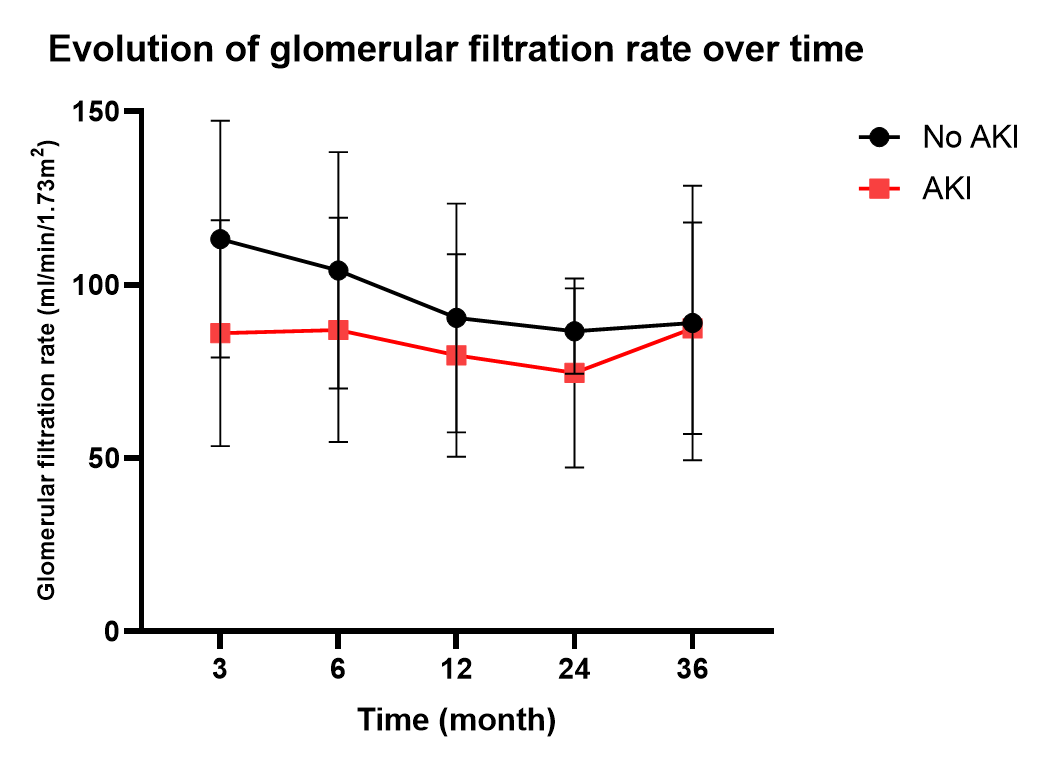


**Figure S5. Bootstrapping analysis of the rasburicase’s influence on mortality and remission**


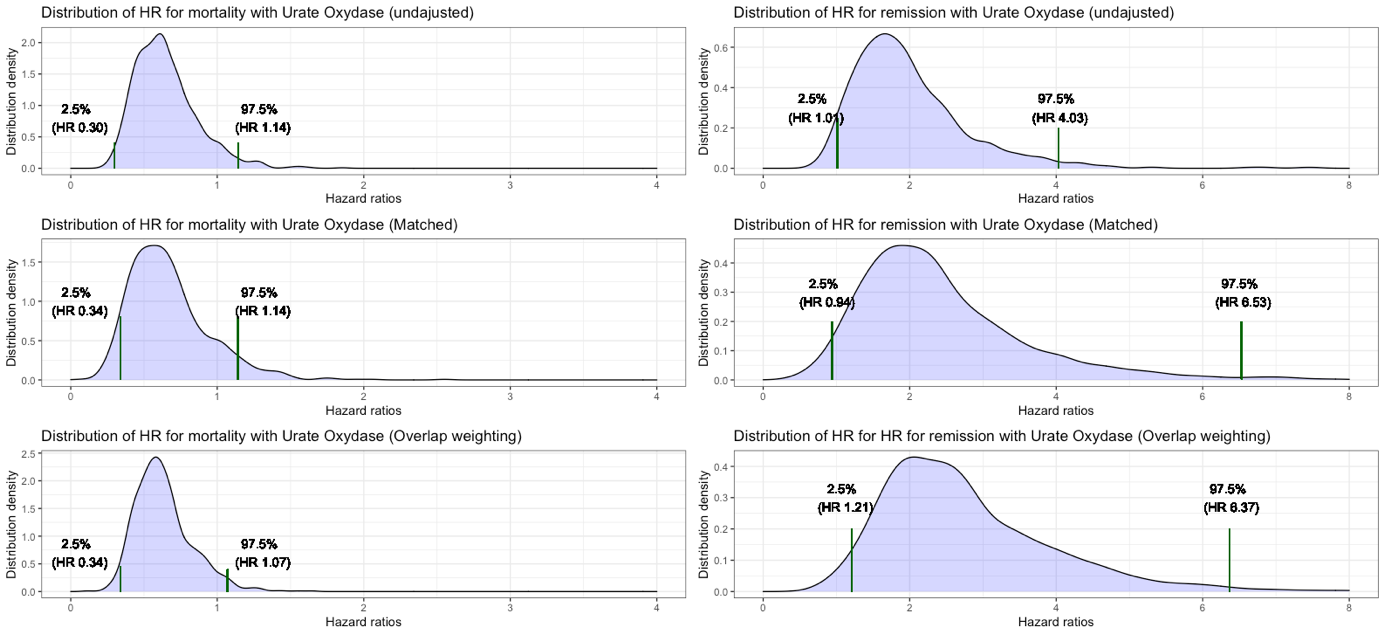

Supplement: Supplementary file 1 — Additional file 1. Additional figures and tables. [file 13613_2022_990_MOESM1_ESM.docx]
